# How childhood maltreatment links to labor values? The mediating role of moral competence and prosocial normative tendency

**DOI:** 10.1186/s40359-022-00833-5

**Published:** 2022-06-22

**Authors:** Yuliang Gu, Xiaomei Chao

**Affiliations:** 1 School of Public Administration, Hunan Normal University, No. 36 Lushan Rd., Yuelu District, Changsha, 410081 Hunan People’s Republic of China; 2 School of Elementary Education, Changsha Normal University, No. 9 Xingshateli Rd., Changsha, 410081 Hunan People’s Republic of China

**Keywords:** Childhood maltreatment, Labor values, Moral competence, Prosocial normative tendency

## Abstract

**Background:**

Labor values are important components of the individual value system and considered to be among the most important values of an individual, especially in China. In studies of values, childhood maltreatment is considered to have an important influence on the formation of individual values. However, there is no previous research about the relationship between childhood maltreatment and labor values. The mechanism of childhood maltreatment on labor values is not clear and requires further study.

**Methods:**

This study intended to investigate the relationship between childhood maltreatment and labor values, and further verify whether moral competence or prosocial normative tendency mediated this correlation. Therefore, 2691 participants were recruited from primary and secondary schools, who completed Labor Values Scale, Childhood Trauma Questionnaire, Moral Competence subscale and Prosocial Norms subscale.

**Results:**

Results revealed the negative correlation between childhood maltreatment and labor values. Importantly, childhood maltreatment also indirectly impacted labor values through moral competence and prosocial normative tendency. It indicated that both moral competence and prosocial normative tendency played a significant mediating role in this relationship. Our findings are valuable for understanding the underlying mechanism between early trauma and values.

**Conclusions:**

Childhood maltreatment has important implications for labor values. Moral competence and prosocial normative tendency mediate between childhood maltreatment and labor values. The results remind us to pay attention to the important influence of childhood maltreatment in the cultivation of labor values, and focus on the role of moral competence and prosocial normative tendency.

Values refer to a preference tendency that meets people’s needs, and are the conceptual system that guides people’s cognitive judgment, choice, and behavioral practice [1–3]. On this basis, some scholars believe that labor values is an important part of the individual value system. Labor values are an abstraction of all subjective evaluations of labor [4, 5]. It is people’s general and fundamental views on labor degree to meet people’s needs, and it is the intrinsic needs of the individual and the labor characteristics and attributes pursued when engaging in activities [6]. There are typical regional and cultural characteristics for values [7, 8]. Similarly, there should also be typical regional and cultural characteristics for labor values.

In the Chinese institutional background directed by Marxism, labor values have special meanings. According to the research of Tan [9], in Chinese culture, labor values refer to the individual’s positive labor attitude, that are, refusing to be lazy, hurting others, self-interest and other negative values. At the same time, labor values encourage people to respect and enjoy the labor process and labor results. Liu [10] elaborated on the connotation of correct labor values from the perspective of labor education. According to his elaboration, correct labor values suggest that “labor is great, laborers and labor results are worthy of respect”. The Chinese government has also discussed what labor values should be established on many occasions. For example, President Xi Jinping believed that positive labor values should be established, of which main contents are honest labor, equal labor, cherishing labor results, love of labor, and distribution on the basis of labor [11]. The above connotations of labor values were also clarified in the research of Chao and Wang [12].

Although labor values are very important [13], the importance of labor education is increasingly being ignored as society changes. Previous studies have shown that an increasing number of young people have less motivation to work [14]. This is mostly because they did not cultivate correct labor values in their early stage. Therefore, it is particularly important to improve the labor values education of the young. In order to this, it is necessary to know factors that may affect young people’s labor values. What factors are noteworthy in labor values education of young people?

These problems remained to be resolved. This is the first research to link childhood maltreatment to adolescent labor values and reveal the potential mechanisms between childhood maltreatment and adolescent labor values. It provided a theoretical and empirical basis for understanding the relationship between adolescents’ early experiences and labor values. At the same time, our research lays the foundation for understanding the connotation of youth labor values in the context of the China’s socialist system, as well as comparing the youth labor values, influencing factors of China and Western countries under different institutional and cultural backgrounds.

## Introduction

### Childhood maltreatment and labor values

Childhood maltreatment refers to caregivers’ actual or potential harmful behavior to children’s physical and mental health [15], including physical abuse, sexual abuse, emotional or psychological abuse and neglect [16]. As an adverse experience in childhood, childhood maltreatment plays an enduring detrimental role in maltreated victims’ physical and mental health [17]. As suggested by literature, negative values might be one of the cognitive outcomes of childhood maltreatment [18, 19]. Values are preferences that meet people’s needs, which form a conception system guiding people’s cognitive judgment, choice and behaviors [20, 21]. Nevertheless, Magdalena [22] has found that it is more difficult for maltreated children to get enough support from their social networks than non-maltreated ones, which hinders the development of positive values. Similarly, Kay and Green [23] has also reported that maltreated adolescents are less likely to form friendly values because of deficits(?) in social cognitive function. It can be concluded that childhood maltreatment is harmful to the formation of positive personal values. To our knowledge, no research has directly examined the relationship between childhood maltreatment and labor values, let alone the underlying mechanism. Investigating these issues is not only helpful for extending our understanding of adverse experiences and negative consequences, but also valuable for developing intervention strategies to reduce the enduring negative effect of this unchangeable adversity (i.e., childhood maltreatment) that shapes labor values. Therefore, this study aims to examine the relation between childhood maltreatment and labor values. In addition, we further explore whether moral competence and prosocial normative tendency serve as mediators in this correlation.

### The mediating role of moral competence

The ability to judge the validity and rationality of certain social phenomena through cognitive control and moral judgment is called moral competence [24]. As a cognitive ability, moral competence may be undermined by unhealthy developmental environment. For instance, Zeng et al. [25] have suggested that individuals who grew up in maltreated environment are more likely to judge themselves negatively. In addition to causing biased judgment toward self-value, maltreated experience is also harmful for social cognition and might cause defect in moral judgment [23, 26]. Thus, childhood maltreatment is an environmental risk factor that might impair moral competence, which might further affect labor values. Though value is relatively stable, it is actually an open and dynamic system that allows changes and adjustments [27]. Identification and self-consistency maintenance [28] are considered to contribute to the changes of values, both of which reflect moral judgement and evaluation to some degree. For example, self-identity and moral judgment are believed to impact the representation of values [29] Additionally, Mars et al. [30] have found that the formation and development of values may be accompanied by cognitive judgment and moral evaluation. These studies imply that moral judgement and evaluation components of moral competence have potential impacts on values, and thus labor values are presumed to be affected by moral competence as well. Based on evidence shown before, it is hypothesized that childhood maltreatment weakens moral competence, through which further negatively influences the formation of positive labor values.

### The mediating role of prosocial normative tendency

Prosocial normative tendency is the probability of persons’ abidance by or being persuaded by social norms. Values are the core structure of socialization process [31], which might be influenced by social norms’ persuasion and social pressure [32]. Pincus et al. [33] have found that the formation and persistence of personal values can be impacted by the motivation to comply with “norms” in peer groups. It is also believed that persuasion could prompt individuals to reevaluate their values, making the change of values possible [34]. Through persuasion, people can realize the unreasonable and absent aspects of the original values and this provides an opportunity for shaping more positive values [35, 36]. Therefore, those who have prosocial normative tendency have increased likelihood to form positive labor values because of their tendency to follow social norms. However, this desired tendency may be harmed by childhood maltreatment, which is an early traumatic and adverse experience. For example, Maughan et al. [37] have found the majority of maltreated children have difficulty in regulating emotions, which makes them less likely to be accepted by peers and internalized social norms [38]. Previous studies have also demonstrated that people with a history of being maltreated have decreased possibility of detecting social norms [39, 40]. Therefore, we hypothesized that childhood maltreatment was detrimental for the formation of prosocial normative tendency, which in turn played a negative effect on labor values.

Based on the above evidence, this study focused on the impact of childhood maltreatment on labor values, and intended to explore the mediating roles of moral competence and prosocial normative tendency. Accordingly, the hypotheses of this research included the following. H1: childhood maltreatment had a negative influence on labor values. H2: childhood maltreatment negatively affects the individual’s ability to distinguish between right and wrong moral competence, which in turn affects the individual’s formation of positive labor values. H3: childhood maltreatment hinders the individual’s prosocial normative tendency development, which in turn affects the individual’s formation of positive labor values.

## Participants and methods

### Participants

Our research uses data from an ongoing project: Labor and Individual Social Development. Part of the data has already been used in previous article [41]. A total of 2749 participants were surveyed. Participants were primary school children and adolescents from two primary schools, two junior high schools and one high school in Mainland China. After receiving the collected questionnaires, we deleted 58 when sorting out the questionnaire data. Our criteria for deleting the questionnaire are as follows: Firstly, before the survey, the principle of voluntary participation in the survey was emphasized. Therefore, in the process of filling out the questionnaires, some people withdrew and did not answer all the questionnaires. Secondly, the answers to the questionnaires filled out by some people had obvious regularity. For example, for 10 consecutive questions, the respondents have chosen the same answer “3”. Then, for 10 consecutive questions, the respondents chose the same answer “2”. Thirdly, when some people fill in the questionnaires, they write some symbols that are not related to the questionnaires. This made the questionnaires they answered ambiguous, and it was difficult to judge the answers they chose. For this case, we define it as: “intentional” nonconforming data. In the end, the effective recovery rate of our questionnaire is 97.89%. 2691 valid questionnaires (1504 from boys and 1187 from girls) remained after deleting all the invalid questionnaires. The sample included the upper grades students of elementary school (fourth, fifth, and sixth grade, 1303 participants), junior high school students (673 participants) and high school students (715 participants). The average age of this sample is 12.50 ± 2.00. Ethics approval for this study was obtained from authors’ organization.

### Measures

#### Labor values Scale

Labor Values Scale (LVS) developed by Chao and Wang [12] based on groups of senior primary school students aged 8–12 was employed to evaluate labor values. This scale consists of 15 items scored from 1 (totally disagree) to 5 (totally agree), including five dimensions (i.e., division value according to labor, honest, equal, cherishing and loving labor value). A sample item is “I enjoy the process of labor”. Higher total scores indicate more positive labor values. At the same time, a study by Chao and Gu [41] confirmed that the scale was also applicable to junior high school students and high school students aged 12–18. In our study, The Cronbach’s α is 0.832.

#### Childhood trauma questionnaire

Childhood Trauma Questionnaire (CTQ) designed by Bernstein et al. [42] and revised by Nina and Xiang [43] was applied to evaluate the situation of childhood maltreatment in age 8–18. The CTQ includes 23 items rated on a 5-point scale (1 = never, 5 = always), and an example item is “My family threatened me in my childhood”. This scale consists of physical abuse, physical neglect, emotional abuse and emotional neglect subscale. The Cronbach α coefficient of the whole scale is 0.898.

#### Moral competence subscale and prosocial norms subscale

The Clear and Positive Identity Subscale developed by Shek et al. [24] was adopted, which contained 15 subscales about the positive development of adolescents. Items of Moral Competence subscale (MC) and Prosocial Norms subscale (PN) were used to measure moral competence and prosocial normative tendency, respectively. Both of the two scales have three items. “I have high moral requirements for my behavior” is an example of MC and “I care about the unfortunate people in society” is an example of PN. All items were scored from 1 (strongly disagree) to 6 (strongly agree). The Cronbach α coefficients of MC and PN are 0.716 and 0.667 respectively.

#### Data analysis

Data analysis is divided into three steps: In the first step, we use Amos 20.0 to establish a measurement model to explore whether each observed variable can represent a latent variable; The second step is to construct a structural model. That is, early abuse as an independent variable, labor values as a dependent variable, and MC and PN as an intermediary to establish a full model; In the third step, we use bootstrap estimation procedure to detect the significance of the intermediary. According to a study by Byrne [44], comparative fitting index (CFI), approximate root mean square error (RMSEA) and standardized root mean square residual (SRMR) were used as fitting indices to evaluate the model fitness.

## Results

### Measurement model

4 latent variables (i.e., labor values, moral competence, prosocial normative tendency and childhood maltreatment) and 15 observation variables are contained in the measurement model. The results have shown the data fits this model well (χ^2^_(84, 2691)_ = 1039.83, *p* < 0.001; RMSEA = 0.065: SRMR = 0.051: CFI = 0.926). And all latent variables are well represented by observed variables because the factor loads of all latent variables are significant (*p* < 0.001). In addition, Table 1 has shown the correlations among four latent variables and demographic factors, and all latent variables are significantly correlated with each other.

**Table 1:** Descriptive statistics and bivariate correlations for all measures

	*M*	*SD*	1	2	3	4	5	6	7
1. Gender			1						
2. Grade			.03	1					
3. Age	12.50	2.00	.06^**^	.91^***^	1				
4. Labor values	59.03	8.65	− .06^**^	− .16^***^	− .17^***^	1			
5. Moral competence	13.42	2.87	− .07^***^	− .03	− .05^*^	.49^***^	1		
6. PNT	14.73	2.60	.11^***^	− .03	− .04^*^	.53^***^	.52^***^	1	
7. CM	32.87	11.89	.02	− .15^***^	− .15^***^	− .23^***^	− .16^***^	− .21^***^	1

*PNT* Prosocial normative tendency; *CM* Childhood maltreatment

^*^p < .05, ^**^p < .01, ^***^p < .001

### Structural model

**Fig. 1:**
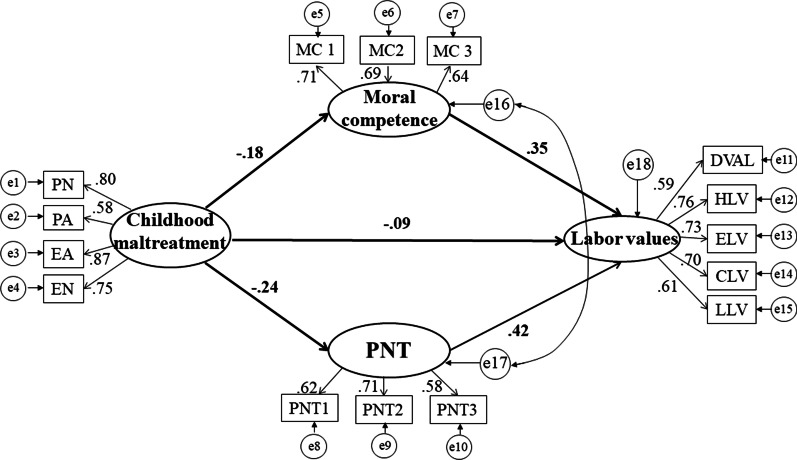
Structural model (N = 2691). *Note*: All path coefficients are significant at the level of .001. PNT = Prosocial normative tendency; CM = Childhood maltreatment. PA, PN, EA and EN represents physical abuse, physical neglect, emotional abuse and emotional neglect. DVAL, HLV, ELV, CLV and LLV represents division value according to labor, honest labor value, equal labor value, cherishing labor value and loving labor value. MC1, MC2, MC3 are items of Moral Competence subscale (MC) and PNT1, PNT2, PNT3 are items of Prosocial Norms subscale

Firstly, childhood maltreatment is related to labor values directly (r = − 0.25, p < 0.001) when mediators are absent. Then a model with mediators of moral competence and prosocial normative tendency are has been built (Model 1). The fitting degree of this model is unsatisfactory: χ^2^_(85, 2691)_ = 1824.47, *p* < 0.001; RMSEA = 0.383; SRMR = 0.106; CFI = 0.865. Therefore, we modified this model by linking errors of two mediators according to modification indices and theoretical relationships (Model 2, see Fig. 1). The Model 2 has shown good fitness (χ^2^_(84, 2691)_ = 1039.83, *p* < 0.001; RMSEA = 0.065; SRMR = 0.051; CFI = 0.926) and thus this model served as structural model in this study.

Bootstrap estimation method (Bootstrap = 1000) is further used to test whether the mediating paths are significant. And results indicate that childhood maltreatment indirectly impacts labor values through moral competence and prosocial normative tendency (see Table 2).

**Table 2:** Standardized indirect effect and 95% confidence intervals

Paths	Estimate	Lower	Upper
CM → moral competence → labor values	− .09	− .12	− .06
CM → PNT → labor values	− .12	− .15	− .08

The mediating effect of a pathway is significant if the 95% confidence interval does not include 0.

### Gender differences

We further examined the gender differences of structural model. Firstly, gender differences in every variable were tested. And results have shown boys score higher than girls in prosocial normative tendency and lower in moral competence and labor values, while there is no statistical difference in childhood maltreatment.

Secondly, gender differences in all path coefficients were tested by using multi-group analysis. After limiting the factor loadings, error variances and structural covariances to be equal, and two models were established following Byrne [44]. The first one constrained all path coefficients in different genders to be equal (constrained structural paths) while the other did not (unconstrained structural paths). Both models fitted data well and they were not significantly different (∆χ^2^_(5, 2691)_ = 9.47, *p* > 0.05). Since χ^2^ value could be affected by the sample size, critical ratios of differences (CRD) were also used to determine the cross-gender stability of Model 2. Two parameters were considered different if the absolute value of CRD was greater than 1.96. And no path coefficients were different in different genders (CRD _CM→MC_ = -1.53; CRD _MC→LV_ = 1.34; CRD _CM→LV_ = 1.03; CRD _CM→PNT_ = -0.08; CRD _PNT→LV_ = -0.07). Based on these, there existed no significant gender difference in the structural model, indicating the cross-gender stability of this model.

### Grade differences

We also tested the grade differences of structural model. First, we examined results in grade differences and found students in lower grades scored higher in childhood maltreatment and labor values, while no grade differences in moral competence and prosocial normative tendency.

Then, grade differences in all path coefficients were also examined using multi-group analysis. Similar to the examination of gender differences, we limited the same parameters to be equal and then established two models (i.e., constrained structural paths and unconstrained structural paths). Though these models were significantly different (∆χ^2^_(10, 2691)_ = 21.74, *p* = 0.02), the results of CRD suggested that no path coefficients were different in different grades (primary school students vs. junior school students: CRD_CM→MC_ = 0.62; CRD_MC→LV_ = − 0.94; CRD_CM→LV_ =  − 0.81; CRD_CM→PNT_ = 1.31; CRD_PNT→LV_ = 0.27; primary school students vs. high school students: CRD_CM→MC_ = 0.53; CRD_MC→LV_ = − 1.46; CRD _CM→LV_ = − 0.63; CRD_CM→PNT_ = 0.89; CRD_PNT→LV_ = 0.57; junior school students vs. high school students: CRD_CM→MC_ = − 0.09; CRD_MC→LV_ = − 0.49; CRD_CM→LV_ = 0.16; CRD_CM→PNT_ = − 0.27; CRD_PNT→LV_ = 0.25). These results indicated there existed no significant grade difference in the structural model.

## Discussion

The present research verified the direct correlation between childhood maltreatment and children’s labor values, and further examined the mediating roles of moral competence or prosocial normative tendency. Results revealed that childhood maltreatment was negatively correlated with labor values. In addition, childhood maltreatment indirectly affected labor values through the mediating effect of moral competence and prosocial normative tendencies. These findings suggest that moral competence and prosocial normative tendency are mediators between childhood maltreatment and labor values for the first time, providing theoretical guidance for inhibiting the influence of early traumatic experience on negative values from the perspective of cognitive ability and social tendency. In addition, for the impact of childhood maltreatment on labor values, there is no significant difference between different gender and grade groups on different paths.

Hypothesis 1 has been supported by the finding of the negative correlation between childhood maltreatment and labor values, corresponding with the evidence that maltreated children are less likely to develop positive values [45]. Education, especially family education from parents, is an important factor in the formation of children’s values [46]. Childhood maltreatment builds an abnormal family education environment for children, which is detrimental to the cultivation of their labor values [47]. Therefore, it is difficult for children to form a positive self-evaluation and self-awareness in the unfavorable family environment of childhood maltreatment [48], which hinders their normal interpersonal interaction [49]. And negative interpersonal environment is detrimental to cultivate labor values. Therefore, childhood maltreatment is negatively related to labor values from the perspective of rational cognition and social interaction.

Interestingly, this study has found that moral competence is a mediator between childhood maltreatment and children’s labor values, which supports hypothesis 2. Previous studies have also reported that childhood maltreatment does harm to individuals’ attitudes or behaviors through undermining their moral competence [50, 51]. Children who have experienced maltreatment usually have defects in social cognitive function and some prejudice in value judgment [23]. Insufficient ability in value judgment of maltreated children is reflected in low moral competence in this study, indicating that it is relatively more difficult for them to make reasonable moral judgments on social phenomenon. Nevertheless, moral competence is important for cultivating children’s labor values. The formation and development of values are series of processes including concept judgment and decision-making [30], and the concept of right and wrong as well as moral competence is critical for shaping values. People with high cognitive control and moral judgment ability are more likely to keep positive and correct values and insist to them [52, 53]. Conversely, people with poor moral competence have deficits in cognitive control and moral judgment, so they are less likely to form or maintain positive values. Therefore, people tend to have negative labor values if their moral competence is insufficient. In a word, childhood maltreatment undermines moral competence, which in turn makes it more difficult for people to form positive and correct labor values.

Similarly, the hypothesis 3 has also been confirmed that prosocial normative tendency mediates the correlation between childhood maltreatment and labor values. It may be because maltreated individuals are more prone to feel angry, and chronic anger further makes them more likely to engage in aggressive behaviors and form antisocial personality [54], which are reveal difficulties in accepting and abiding to social norms. Moreover, maltreated children are less likely to recognize and comply with social norms [39], which also suggests that childhood maltreatment experience is detrimental for developing prosocial normative tendency. However, this tendency plays an important role for people to accept persuasion from others, which further reduces the possibility of modifying inappropriate conceptions or develop positive ones. Values consist of systematic conceptions [1, 2], and persuasion is a critical influencing factor of individual values [55]. It is believed that logical persuasion and restrictions of social norms can cultivate values by influencing cognitive process [35]. And the reason why social norms shapes values may be that conceptions are reevaluated and incorporated to existing value system [36]. Based on above discussions, it can be understood that maltreated children have reduced probability of recognition and abidance to social norms (i.e., prosocial normative tendency), which further makes them tend to form negative labor values.

For various reasons, our research still has some limitations. Firstly, our respondents are from China. As a socialist country, Chinese individuals have attached great importance to the education of labor values since childhood. Compared to China, other countries may place emphasis on labor values education in a very different way. Therefore, the relationship between childhood maltreatment and labor values in different countries needs further comparison. Secondly, our study is based on the analysis of cross-sectional data. It is difficult to determine the temporal relationship between variables when using cross-sectional data to explore the mediation relationship. Therefore, our study still has some limitations in the inference of the results. Thirdly, retrospective reporting should be acknowledged as limitation. In the original questionnaire, the instructions mainly asked to review the experience of abuse before the age of 16, which is regarded as the dividing line of early abuse experiences. In our project implementation, we hope that we can cover the entire population of senior elementary school students, junior high school students and high school students close to K12. Therefore, we cannot define the period of early abuse with the dividing line of age of 16, but as “childhood”. This will inevitably lead to the problem that some samples in lower grades are suffering the same maltreatment as the so-called “children”. And with age reduces, it is easier for the participants to have this problem. Even if we ask high school students to fill in the relevant questionnaires that uses the age-of-16 standard of the original questionnaire, it is still difficult to rule out maltreatment that have continued since childhood. Due to all these limitations of our study, we remind readers to be more objective when reading our manuscript.

## Data Availability

The datasets analysed during the current study are not publicly available due to that the project covered in this manuscript is still ongoing, but are available from the corresponding author on reasonable request.
